# Severe sinus bradycardia associated with favipiravir in a COVID‐19 patient

**DOI:** 10.1002/ccr3.4566

**Published:** 2021-08-06

**Authors:** Mhd Baraa Habib, Mohamed Elshafei, Alaa Rahhal, Mouhand F. H. Mohamed

**Affiliations:** ^1^ Department of Internal Medicine Hamad Medical Corporation Doha Qatar; ^2^ Department of Pharmacy Hamad Medical Corporation Doha Qatar

**Keywords:** drug‐associated, favipiravir, severe bradycardia, sinus rhythm

## Abstract

The antiviral medication “favipiravir” should be considered as a possible cause of unexplained sinus bradycardia.

## Graphical abstract

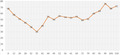

## 1 INTRODUCTION

Favipiravir is an antiviral medication which can cause adverse effects such as gastrointestinal upset, but rarely cardiac side effects. Here, we present a case of a middle‐aged lady, who developed asymptomatic severe sinus bradycardia with a heart rate 30, which was attributed to favipiravir. Bradycardia resolved after discontinuing the medication.

Favipiravir is an oral purine base analog that inhibits RNA‐dependent RNA polymerase of RNA viruses, which eventually prevent viral replication. Favipiravir was previously approved for the management of the new or reemerging pandemic influenza infection in Japan in 2014.^1^ It also has been recently used in the treatment of coronavirus disease 2019 (COVID‐19).^1^ Some published studies showed a good therapeutic response in COVID‐19 patients in terms of viral clearance and disease progression.^2^ On the contrary, adding favipiravir to the treatment protocol has not reduced the number of intensive care unit (ICU) admissions, the need for mechanical ventilation, or in‐hospital mortality compared with lopinavir/ritonavir regimen.^3^

The suggested dose of favipiravir for COVID‐19 patients is 1600 mg loading dose twice on day one, followed by a maintenance dose of 600 mg twice daily for additional six days.^2^ It is generally a safe medication but can cause adverse effects such as gastrointestinal (GI) upset, transaminitis, and rarely cardiac side effects. To the best of our knowledge, favipiravir has not been reported to cause significant bradycardia.^1^

## 2 CASE PRESENTATION

**FIGURE 1:**
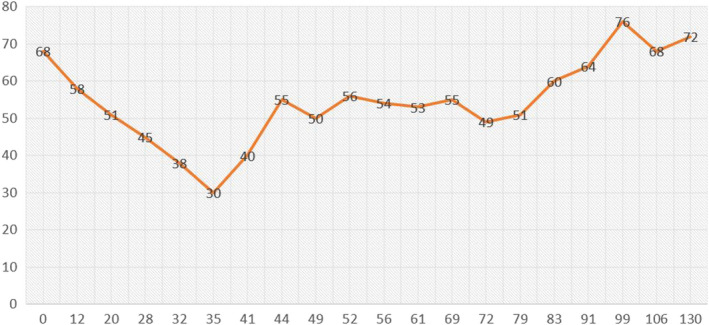
The patient's pulse rate (beat/minute) (*Y*‐axis) over 130 h (*X*‐axis) during the hospital stay

**FIGURE 2:**
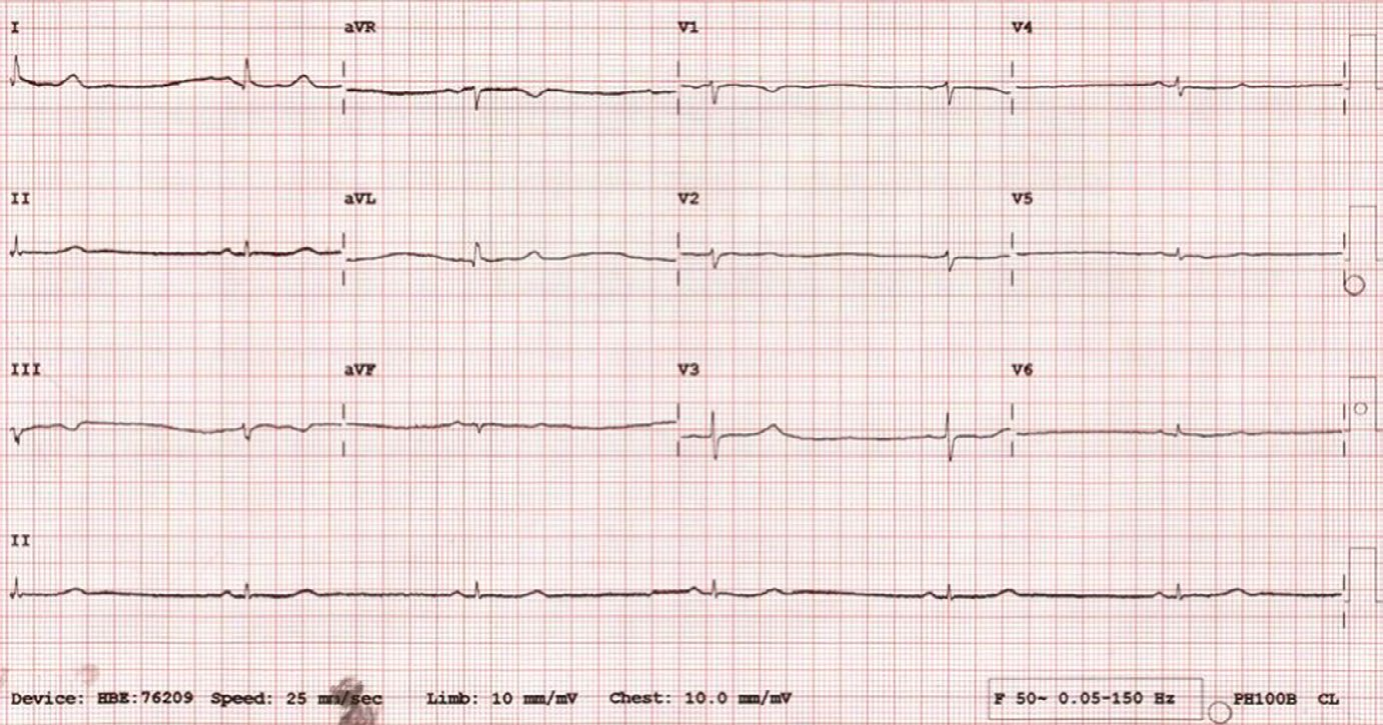
ECG during favipiravir treatment shows severe bradycardia with heart rate around 33/min

A 52‐year‐old woman, without known chronic illnesses, presented to our hospital with a five‐day history of fever, fatigue, dry cough, and progressive shortness of breath. Chest X‐ray showed bilateral patchy infiltrates concerning viral pneumonia. Polymerase chain reaction (PCR) of the nasopharyngeal swab was positive for SARS‐COV‐2. She was admitted as a case of COVID‐19 pneumonia. The patient was started on COVID‐19 pneumonia treatment according to the local guidelines in Qatar, which contained oral favipiravir 1600 mg twice per day for 1 day then 600 mg twice per day, oral doxycycline 100 mg twice daily, intravenous dexamethasone 8 mg daily, paracetamol as needed for fever, and metoclopramide as needed for nausea (received only one dose on admission). During hospitalization, the patient became afebrile. The next day (day 2 post‐admission), the patient developed bradycardia, and her heart rate started to drop as shown in Figure 1, reaching 30/min, although she was awake and anxious about the heart rate number and not able to sleep. However, the patient denied any bradycardia‐related symptoms. Electrocardiogram (ECG) showed sinus bradycardia (Figure 2). Serial troponin tests were negative, and other laboratory tests including electrolytes were generally within the normal range as demonstrated in Table 1. As the patient was asymptomatic, she was kept on cardiac monitoring under close observation. We suspected that bradycardia is a side effect of her medications. Dexamethasone and metoclopramide were suspended. However, her heart rate remained between 40 and 50/min. Favipiravir was suspended on day four due to elevated transaminases, and dexamethasone was resumed. Subsequently, we noticed that her heart rate started to increase gradually until it reached 72/min on day six, then stabilized between 60 and 80 beats/min. Later, the patient had Holter monitoring which was unremarkable for any arrhythmia or conduction abnormalities. Echocardiography and stress test were normal as well. Hence, the diagnosis was sinus bradycardia probably induced by favipiravir.

**TABLE 1:** Blood tests during hospitalization

Detail	Value w/Units	Normal range
Free thyroxine	13.2 pmol/L	11.6–21.9
Urea	4.3 mmol/L	2.8–8.1
Creatinine	51 umol/L	44–80
Sodium	141 mmol/L	136–145
Potassium	4.1 mmol/L	3.5–5.1
Troponin‐T high sensitivity	6 ng/L	3–10
Thyroid stimulating hormone	0.68 mIU/L	0.30–4.20
White blood cell count	4.6 ×10^3^/ul	4.0–10.0
Hemoglobin	13.6 gm/dl	12.0–15.0
Adjusted calcium	2.22 mmol/L	2.15–2.50
Magnesium	0.80 mmol/L	0.66–1.07
Bilirubin T	5 umol/L	0–21

## 3 DISCUSSION

Sinus bradycardia is defined as a heart rate less than 60 beats per minute associated with a normal P wave. Bradycardia can be asymptomatic or comes with symptoms including chest pain, dyspnea, fatigue, dizziness, or syncope.^4^ Many conditions can be associated with sinus bradycardia, for example, ischemic heart disease, hypothyroidism, hyperkalemia, amyloidosis, and intracranial hypertension. Additionally, medications such as beta‐blockers, calcium channel blockers, digoxin, and ivabradine are common causes of bradycardia as well.^4^ The treatment for asymptomatic sinus bradycardia is generally not required. However, treating the underlying cause is the mainstay of management.^5^ Sinus bradycardia was noticed in a few reports of severe COVID‐19 pneumonia.^6^, ^7^, ^8^ The inhibitory effect of the virus on sinus node activity, hypoxia, and inflammatory damage to the nodal cells were suggested as possible mechanisms behind sinus bradycardia in patients with severe COVID‐19 pneumonia.^6^ Common adverse effects related to favipiravir include gastrointestinal upset, diarrhea, hyperuricemia, transaminitis, and neutropenia.^1^ Cardiac side effects (eg, chest pain) were rarely reported.^9^ Drug‐induced bradycardia is very common,^5^ however, bradycardia as a side effect of favipiravir is rare and was mentioned in a preliminary report of favipiravir observational study in Japan with an event rate of 0.1% only.^10^

In our case, the patient developed severe sinus bradycardia with a heart rate nadir of 30 beats/minute a few hours after receiving the loading dose of favipiravir. Her heart rate slightly increased but remained around 40/min when she was kept on the maintenance dose of favipiravir. Although bradycardia was reported as a rare adverse effect of dexamethasone^11^ and metoclopramide,^12^ the patient's heart rate did not normalize when they were suspended. Furthermore, heart rate improved and reached normal limits when favipiravir was held, and dexamethasone resumed. Naranjo Scale^13^ showed a probable relationship (score of 6) between the patient's favipiravir intake and bradycardia. Because of ethical considerations, we could not rechallenge the patient with favipiravir despite its proposed antiviral efficacy.

Given the time‐relation between favipiravir initiation and bradycardia, the persistent bradycardia while the patient was receiving favipiravir, as well as the improvement of heart rate after suspending favipiravir, we attributed the patient's bradycardia to favipiravir use. After discharge, the patient was followed in the cardiology clinic. She underwent an echocardiogram, Holter monitoring, and stress test which all came back unremarkable for any cardiac pathology. The former negative investigations also support the diagnosis of transient bradycardia induced by favipiravir. Further studies may be needed to clarify the underlying mechanisms.

## 4 CONCLUSION

This case describes favipiravir‐associated significant bradycardia, which could be dose‐dependent. Favipiravir should be considered a possible cause of unexplained sinus bradycardia. Further large studies are needed to confirm this observation.

## CONFLICT OF INTEREST

The authors report no conflict of interest.

## AUTHOR CONTRIBUTIONS

MBH: involved in conceptualization, and writing the original draft. ME and AR: participated in literature review and reviewing the manuscript. MM: involved in supervision and writing the review and editing.

## PATIENT’S CONSENT

Due to the COVID‐19 situation and its impact on direct patient contact, only verbal consent was obtained to publish this case.

## Data Availability

The data that support the findings of this study are available from authors, MBH and MM, upon reasonable request.
